# Suppression of Expression of Heat Shock Protein 70 by Gefitinib and Its Contribution to Pulmonary Fibrosis

**DOI:** 10.1371/journal.pone.0027296

**Published:** 2011-11-09

**Authors:** Takushi Namba, Ken-Ichiro Tanaka, Tatsuya Hoshino, Arata Azuma, Tohru Mizushima

**Affiliations:** 1 Graduate School of Medical and Pharmaceutical Sciences, Kumamoto University, Kumamoto, Japan; 2 Division of Respiratory, Infection and Oncology, Department of Internal Medicine, Nippon Medical School, Tokyo, Japan; 3 Department of Analytical Chemistry, Faculty of Pharmacy, Keio University, Tokyo, Japan; University of Nebraska Medical Center, United States of America

## Abstract

Drug-induced interstitial lung disease (ILD), particularly pulmonary fibrosis, is of serious clinical concern. Gefitinib, a tyrosine kinase inhibitor of the epidermal growth factor receptor (EGFR), is beneficial as a drug for treating non-small cell lung cancer; however, this drug induces ILD and the molecular mechanisms underpinning this condition remain unclear. We recently reported that expression of heat shock protein 70 (HSP70) protects against bleomycin-induced pulmonary fibrosis, an animal model of pulmonary fibrosis. In this study, we have examined the effects of drugs known to induce ILD clinically on the expression of HSP70 in cultured lung epithelial cells and have found that gefitinib has a suppressive effect. Results of a luciferase reporter assay, pulse-labelling analysis of protein and experiments using an inhibitor of translation or transcription suggest that gefitinib suppresses the expression of HSP70 at the level of translation. Furthermore, the results of experiments with siRNA for Dicer1, an enzyme responsible for synthesis of microRNA, and real-time RT-PCR analysis suggest that some microRNAs are involved in the gefitinib-induced translational inhibition of HSP70. Mutations in the EGFR affect the concentration of gefitinib required for suppressing the expression of HSP70. These results suggest that gefitinib suppresses the translation of HSP70 through an EGFR- and microRNA-mediated mechanism. *In vivo*, while oral administration of gefitinib suppressed the pulmonary expression of HSP70 and exacerbated bleomycin-induced pulmonary fibrosis in wild-type mice, these effects were not as distinct in transgenic mice expressing HSP70. Furthermore, oral co-administration of geranylgeranylacetone (GGA), an inducer of HSP70, suppressed gefitinib-induced exacerbation of bleomycin-induced pulmonary fibrosis. Taken together, these findings suggest that gefitinib-induced exacerbation of bleomycin-induced pulmonary fibrosis is mediated by suppression of pulmonary expression of HSP70 and that an inducer of HSP70 expression, such as GGA, may be therapeutically beneficial for the treatment of gefitinib-induced pulmonary fibrosis.

## Introduction

Interstitial lung disease (ILD), in particular interstitial pneumonia associated with pulmonary fibrosis, is a devastating chronic lung condition with poor prognosis. Pulmonary fibrosis progresses insidiously, with acute exacerbation of interstitial pneumonia being a highly lethal clinical event [1], [2]. Although most cases of pulmonary fibrosis are idiopathic, some are due to drug side effects (drug-induced pulmonary fibrosis). For example, the anti-tumour drugs gefitinib and imatinib, as well as anti-rheumatoid arthritis drugs such as leflunomide, are known to induce ILD (pulmonary fibrosis). This is cause for serious clinical concern, as it restricts the therapeutic use of these drugs [3], [4], [5]. Unfortunately, the etiology of drug-induced ILD (pulmonary fibrosis) is not yet understood and, as a result, an appropriate animal model has not yet been established. Understanding the mechanism governing drug-induced ILD (pulmonary fibrosis) and developing a viable animal model are therefore important to establish not only a clinical protocol for its treatment but also an assay system that will facilitate screening in order to eliminate candidate drugs with the potential to produce this type of side effect. Bleomycin-induced pulmonary fibrosis in animals mimics some characteristics of human pulmonary fibrosis [6]. We recently reported that leflunomide exacerbates bleomycin-induced pulmonary fibrosis, proposed that this model is a suitable animal model for drug-induced ILD, and suggested that this exacerbation is mediated by epithelial-mesenchymal transition (EMT) of lung epithelial cells [7]. However, the molecular mechanisms underpinning ILD (pulmonary fibrosis) induced by drugs other than leflunomide remain unclear.

Pulmonary fibrosis is induced by repeated epithelial cell damage by reactive oxygen species (ROS) and other stressors and abnormal wound repair and remodelling, resulting in abnormal deposition of extracellular matrix (ECM) proteins, such as collagen. In addition to the increase in transforming growth factor (TGF)-β1 [8], an increase in the level of lung myofibroblasts has been suggested to play an important role in pulmonary fibrosis [9]. It was previously believed that the sole origin of myofibroblasts is peribronchiolar and perivascular fibroblasts that transdifferentiate into myofibroblasts [10]. However, recently, it was revealed that some of the lung myofibroblasts in pulmonary fibrosis patients originate from lung epithelial cells via EMT [11], [12], [13], [14].

Gefitinib, a tyrosine kinase inhibitor of the epidermal growth factor receptor (EGFR), is a new molecular target agent for the treatment of patients with advanced non-small cell lung cancer who fail to respond to chemotherapy [15]. Furthermore, recent clinical studies have shown that this drug is particularly effective for patients with EGFR mutations, which causes constitutive activation of EGFR-dependent intracellular signal transduction [16], [17]. Although gefitinib has been recognised as relatively safe based on data from clinical trials, post-marketing surveillance of patients prescribed with gefitinib in Japan has revealed that 6.8% of patients developed interstitial pneumonia and that, of these, 40% of the patients died [4], [18], [19]. The incidence of gefitinib-induced ILD and its mortality rate are higher in Japan than in Western countries [20], [21]. However, the mechanism governing gefitinib-induced ILD (pulmonary fibrosis) and the reason for this ethnic difference is unknown. Furthermore, contradictory results have been reported regarding the effect of gefitinib on bleomycin-induced pulmonary fibrosis in animals (prevention and exacerbation) [22], [23] and the mechanisms governing these phenomena are unknown.

Different stressors induce cells to express heat shock proteins (HSPs) through transcriptional regulation mediated by a transcription factor, heat shock factor 1 (HSF1), and a *cis*-element located in the *hsp* gene promoter, heat shock element (HSE) [24]. HSPs, especially HSP70, expressed in cultured cells protect these cells against a range of stressors, including ROS, by refolding or degrading denatured proteins produced by the stressors (HSPs function as molecular chaperones) [24], [25]. Interestingly, geranylgeranylacetone (GGA), a leading anti-ulcer drug on the Japanese market, has been reported to be a non-toxic HSP-inducer [26], [27]. In addition to the cytoprotective effects of HSP70, its anti-inflammatory effects have been identified recently [28]. We have shown that through the cytoprotective, anti-inflammatory and molecular chaperone activities, both genetic and pharmacologic (by GGA) induction of expression of HSP70 is protective in animal models of various diseases, such as gastric and small intestinal lesions, inflammatory bowel diseases, ultraviolet light-induced skin damage and Alzheimer's disease [29], [30], [31], [32], [33], [34]. Furthermore, we recently reported that bleomycin-induced lung injury, inflammation, fibrosis and dysfunction are suppressed in transgenic mice expressing HSP70 or in GGA-administered wild-type mice. We also suggested that HSP70 plays this protective role through cytoprotective and inflammatory effects and by inhibiting the production of TGF-β1 and TGF-β1-dependent EMT of lung epithelial cells [35].

As a mechanism for the regulation of gene expression, microRNAs (miRNAs) have been paid much attention recently. miRNAs are short non-coding single-stranded RNA species which bind to complementary regions of the 3' untranslated regions (UTRs) of mRNA resulting in repression of translation and/or stimulation of degradation of mRNA. Primary miRNA transcripts are first processed in the nucleus to produce hairpin RNAs (pre-miRNAs), which are then exported into the cytoplasm, where Dicer1 cuts the hairpin to produce miRNAs [36]. Aberrant expression of miRNAs is associated with pathologic conditions, such as cancer, diabetes and fibrosis [36], [37].

In this study, we examined the effect on the expression of HSP70 of drugs known to induce ILD clinically in cultured lung epithelial cells, and found that gefitinib suppresses the expression of HSP70. The results suggest that gefitinib regulates expression of HSP70 at the level of translation through an EGFR- and miRNA-mediated mechanism. We also found that oral administration of gefitinib suppresses pulmonary expression of HSP70 and suggested that this suppression is involved in gefitinib-induced exacerbation of bleomycin-induced pulmonary fibrosis. These results suggest that HSP70 plays an important role in gefitinib-induced ILD (pulmonary fibrosis) and that examination of the effect of drugs on HSP70-expression *in vitro* is useful as a screening system in order to eliminate candidate drugs with the potential to induce ILD.

## Materials and Methods

### Ethics Statement

The experiments and procedures described here were carried out in accordance with the Guide for the Care and Use of Laboratory Animals as adopted and promulgated by the National Institutes of Health, and were approved by the Animal Care Committee of Kumamoto University. Permit numbers or approval ID for this study is C-20–166-R1.

### Chemicals and animals

Paraformaldehyde, fetal bovine serum (FBS), 4-(dimethylamino)-benzaldehyde (DMBA), chloramine T, cycloheximide, SP600125, Orange G, RPMI1640 and DMEM were obtained from Sigma (St. Louis, MO). Bleomycin was purchased from Nippon Kayaku (Tokyo, Japan). An RNeasy kit, miScript miRNA Mimic and HiPerFect were obtained from Qiagen (Valencia, CA), the PrimeScript® 1st strand cDNA synthesis kit was from TAKARA Bio (Ohtsu, Japan), and the iQ SYBR Green Supermix was from Bio-Rad (Hercules, CA). The mirVana miRNA isolation kit and pMIR-REPORT System were purchased from Applied Biosystems (Carlsbad, CA). Antibodies against actin and HSP70 were purchased from Santa Cruz Biotechnology, Inc. (Santa Cruz, CA) and BD Bioscience (San Francisco, CA), respectively. Antibodies against HSP27, HSP47, HSP60 and HSP90 were from Stressgene (San Francisco, CA). The NCode VILO miRNA cDNA synthesis kit and Lipofectamine (TM2000) were obtained from Invitrogen (Carlsbad, CA). A771726, gefitinib, imatinib, amiodarone, L-hydroxyproline, azophloxin and aniline blue were from WAKO Pure Chemicals (Tokyo, Japan). Xylidine ponceau was from WALDECK GmbH & Co. (Muenster, Germany), and Mayer's hematoxylin, 1% eosin alcohol solution, mounting medium for histological examination (malinol) and Weigert's iron hematoxylin were from MUTO Pure Chemicals (Tokyo, Japan). Transgenic mice expressing HSP70 were gifts from Drs. CE Angelidis and GN Pagoulatos (University of Ioannina, Ioannina, Greece) and were crossed with C57BL/6J wild-type mice 10 times to generate the mice used in this study [32].

### Cell culture

A549 and H1975 cells, and PC9 cells were cultured in DMEM and RPMI1640 medium, respectively, supplemented with 10% FBS, 100 U/ml penicillin and 100 µg/ml streptomycin in a humidified atmosphere of 95% air with 5% CO_2_ at 37°C.

### Real-time RT-PCR analysis

Real-time RT-PCR was performed as previously described [38] with some modifications. Total RNA was extracted from cells using an RNeasy kit or mirVana miRNA isolation kit according to the manufacturer's protocol. Samples were reverse-transcribed using a first-strand cDNA synthesis kit or NCode VILO miRNA cDNA synthesis kit. Synthesized cDNA was used in real-time RT-PCR experiments (Chromo 4 instrument; Bio-Rad Laboratories) using iQ SYBR GREEN Supermix, and analyzed with Opticon Monitor Software. Specificity was confirmed by electrophoretic analysis of the reaction products and by the inclusion of template- or reverse transcriptase-free controls. To normalize the amount of total RNA present in each reaction, *actin* or RUN44 cDNA was used as an internal standard. Primers were designed using the Primer3 website or NCode™ miRNA database website.

The primers used were (name: forward primer, reverse primer): *hsp27*: 5'-ccacccaagtttcctcctc-3', 5'-gactgggatggtgatctcgt-3'; *hsp47*: 5'-ccatgttcttcaagccacact-3', 5'-cgtagtagttgtagaggcctgt-3'; *hsp60*: 5'-tttcagatggagtggctgtg-3', 5'-caatgccttcttcaacagca-3'; *hsp70*: 5'-aggccaacaagatcaccatc-3', 5'-tcgtcctccgctttgtactt-3'; *hsp90*: 5'-ggcagaggctgataagaacg-3', 5'-ctggggatcttccagactga-3'; *actin*: 5'-ggacttcgagcaagagatgg-3', 5'- agcactgtgttggcgtacag-3'; miR-146a: 5'-tgagaactgaattccatgggtt-3'; miR-146b-5p: 5'-gtgagaactgaattccataggct-3'; miR-223*: 5'-gcgtgtatttgacaagctgagtt-3'; miR-561: 5'-cgcaaagtttaagatccttgaagt-3'; miR-449a: 5'-tggcagtgtattgttagctggt-3'; miR-449b: 5'-aggcagtgtattgttagctggc-3'; RUN44: 5'-gagctaattaagaccttcatgttca-3', 5'-cctggatgatgataagcaaatg-3'. For miRNAs, the universal primer in the NCode VILO miRNA cDNA synthesis kit was used as the reverse primer.

We searched for miRNAs that potentially bind to the 3' UTR of *hsp70*, using the TargetScan and Segal Lab websites.

### Luciferase assay

DNA fragments of the *hsp70* 3' UTR (from 2169 to 2427) were amplified by PCR and ligated into the Spe*I*-Hin*dIII* site of the *Photinus pyralis* luciferase reporter plasmid (pMIR-REPORT) to generate pMIR/luc/*hsp70* 3' UTR. The pGL-3/HSE plasmid was constructed by inserting HSE just upstream of the *luciferase* gene. The pGL-3/*hsp70*pro plasmid, which was constructed by inserting the *hsp70* promoter into the same region, was generously provided by Dr. Chang EB (University of Chicago). The luciferase assay was performed as described previously [38]. Transfections were carried out using Lipofectamine (TM2000) according to the manufacturer's instructions. Cells were used for experiments after a 24 h recovery period. Transfection efficiency was determined in parallel plates by transfection of cells with a pEGFP-N1 control vector. Cells were transfected with 0.5 µg of one of the *Photinus pyralis* luciferase reporter plasmids (pMIR/luc/*hsp70* 3' UTR, pGL-3/*hsp70*pro or pGL-3/HSE) and 0.125 µg of the internal standard plasmid bearing the *Renilla reniformis* luciferase reporter (pRL-SV40). *Photinus pyralis* luciferase activity in the cell extracts was measured using the Dual Luciferase Assay System and then normalized for *Renilla reniformis* luciferase activity.

### Immunoblotting analysis

Whole cell extracts were prepared as described previously [38]. The protein concentration of the samples was determined by the Bradford method [39]. Samples were applied to polyacrylamide SDS gels, subjected to electrophoresis, and the resultant proteins immunoblotted with their respective antibodies.

### Pulse-chase and pulse-labelling analyses

Pulse-chase and pulse-labelling analyses were carried out as described previously [40], with some modifications. Cells were labelled with [^35^S]methionine and [^35^S]cysteine in methionine- and cysteine-free RPMI1640 medium for 15 min. To chase labelled proteins, cells were washed with fresh complete (with methionine and cysteine) medium three times and incubated in complete medium for 4 or 8 h. HSP70 was immunoprecipitated with its antibody and separated by SDS-polyacrylamide gel electrophoresis, and visualised by autoradiography (Fuji BAS 2500 imaging analyzer).

### Transfection of cells with siRNA or miRNA mimic RNA fragments

The siRNA for Dicer1 and the miRNA mimic RNA fragments for miR-146a and miR-146b-5p were purchased from Qiagen. A549 cells were transfected with these RNAs using HiPerFect transfection reagents according to the manufacturer's instructions. The siRNA (5'-uucuccgaacgugucacgudTdT-3' and 5'-acgugacacguucggagaadTdT-3') was used as a non-specific siRNA.

### Administration of bleomycin, gefitinib and GGA

C57BL/6 mice were maintained under chloral hydrate anesthesia (500 mg/kg) and given one intratracheal injection of bleomycin (1 or 2 mg/kg) to induce fibrosis. Gefitinib (200 mg/kg) was dissolved in 1% methylcellulose and administered orally. GGA (200 mg/kg) was dissolved in 5% arabic gum and 0.06% Tween and administered orally.

### Histological analysis

Lung tissue samples were fixed in 4% buffered paraformaldehyde and embedded in paraffin before being cut into 4 µm-thick sections.

For histological examination, sections were stained first with Mayer's haematoxylin and then with 1% eosin alcohol solution. Samples were mounted with malinol and inspected with the aid of an Olympus BX51 microscope.

For Masson's trichrome staining of collagen, sections were sequentially treated with solution A (5% (w/v) potassium dichromate and 5% (w/v) trichloroacetic acid), Weigert's iron hematoxylin, solution B (1.25% (w/v) phosphotungstic acid and 1.25% (w/v) phosphomolybdic acid), 0.75% (w/v) Orange G solution, solution C (0.12% (w/v) xylidine ponceau, 0.04% (w/v) acid fuchsin and 0.02% (w/v) azophloxin), 2.5% (w/v) phosphotungstic acid, and finally aniline blue solution. Samples were mounted with malinol and inspected with the aid of an Olympus BX51 microscope.

### Hydroxyproline determination

Hydroxyproline content was determined as previously described [41]. Briefly, the right lung was removed and homogenized in 0.5 ml of 5% TCA. After centrifugation, pellets were hydrolyzed in 0.5 ml of 10 N HCl for 16 h at 110°C. Each sample was incubated for 20 min at room temperature after the addition of 0.5 ml of 1.4% (w/v) chloramine T solution and then incubated at 65°C for 10 min after the addition of 0.5 ml of Ehrlich's reagent (1M DMBA, 70% (v/v) isopropanol and 30% (v/v) perchloric acid). Absorbance was measured at 550 nm and the amount of hydroxyproline was determined.

### Statistical analysis

Two-way analysis of variance (ANOVA), followed by the Tukey test or the Student's *t*-test for unpaired results, was used to evaluate differences between more than three groups or between two groups, respectively. Differences were considered to be significant for values of *P*<0.05.

## Results

### Suppression of expression of HSP70 by gefitinib

We first examined the effects of drugs known to induce ILD clinically (A771726 (an active metabolite of leflunomide), amiodarone, gefitinib and imatinib) on the expression of HSP70 in cultured human type II alveolar (A549) cells. As shown in Fig. S1, a decrease in the level of HSP70 was observed in cells treated with gefitinib but not in cells treated with other drugs.

**Figure 1:**
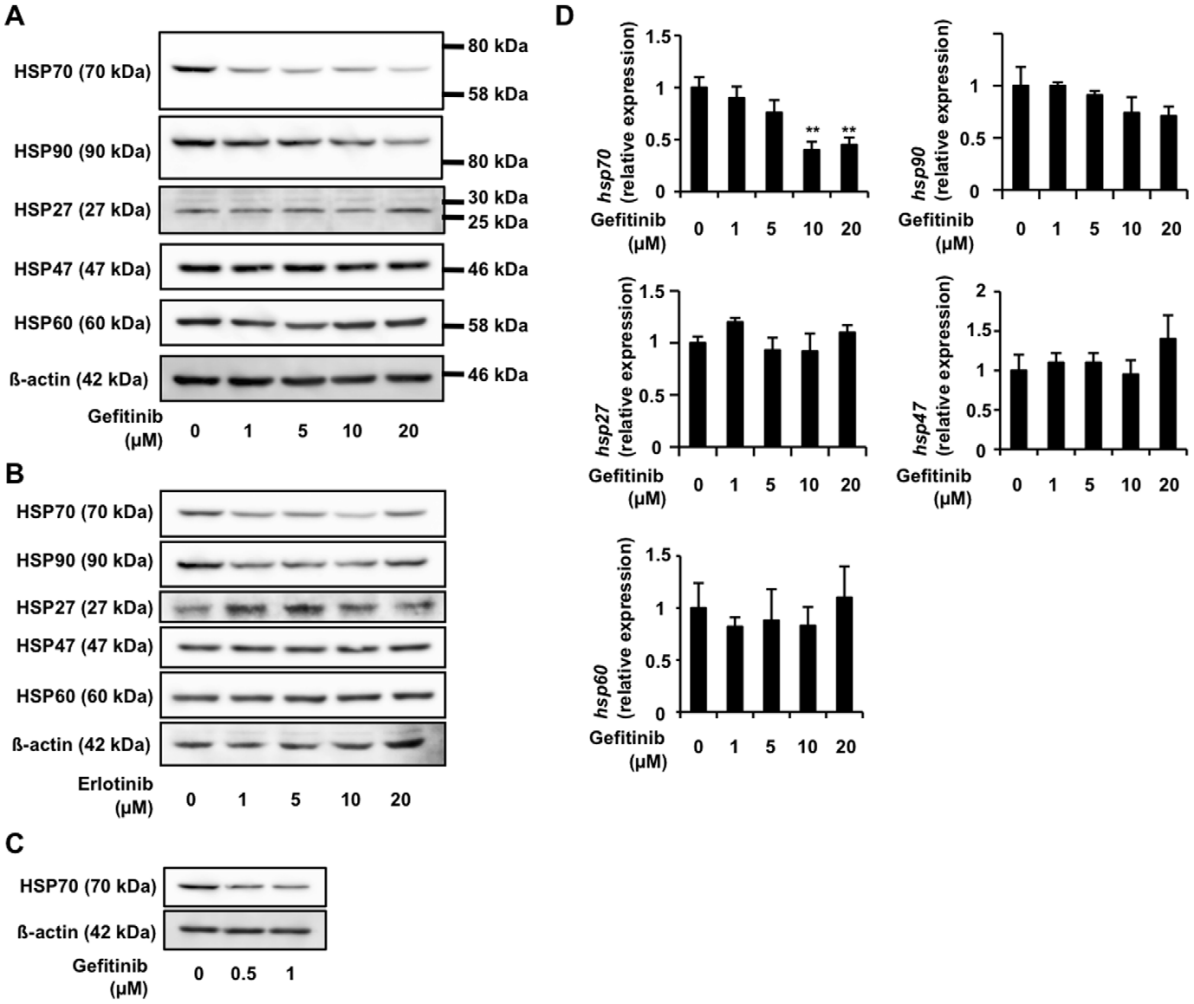
Suppression of expression of HSPs by gefitinib. A549 cells were incubated with the indicated concentration of gefitinib (A, C) or erlotinib (B) for 24 h (A, B) or 12 h (C). Whole-cell extracts were analyzed by immunoblotting with an antibody against HSP70, HSP90, HSP27, HSP47, HSP60 or actin (A, B). Total RNA was extracted and subjected to real-time RT-PCR using a specific primer set for each gene. Values were normalized to the *actin* gene, expressed relative to the control sample (C). Values shown are mean ± S.D. (*n* = 3). **P<0.01.

As shown in Fig. 1A, treatment of cells with gefitinib decreased the level of not only HSP70 but also HSP90 in a dose-dependent manner; however, the decrease was only clearly observed for HSP70 and not for HSP90, at the concentration of 1 µM. In contrast, levels of other HSPs (HSP27, HSP47 and HSP60) were not affected by treatment with gefitinib (Fig. 1A). Erlotinib is another inhibitor of EGFR used clinically [42] and here we found that this drug also decreases the levels of HSP70 and HSP90 (Fig. 1B), suggesting that the inhibitory effect of gefitinib on EGFR is involved in the decrease in the level of HSP.

Real-time RT-PCR analysis revealed that treatment of cells with gefitinib at 1 µM, a concentration determined to be sufficient to decrease the level of HSP70 (Fig. 1A), did not affect the *hsp70* mRNA level; however, this drug decreased the level at concentrations higher than 10 µM (Fig. 1C).

### Suppression of translation of HSP70 by gefitinib

The cellular level of protein is regulated by various factors, such as transcription, mRNA stability, translation and protein stability. With these possibilities in mind, we then examined which of these factors is affected by gefitinib in relation to HSP70. The results in Fig. 1C suggest that 1 µM gefitinib does not affect the transcription and mRNA stability of *hsp70*.

**Figure 2:**
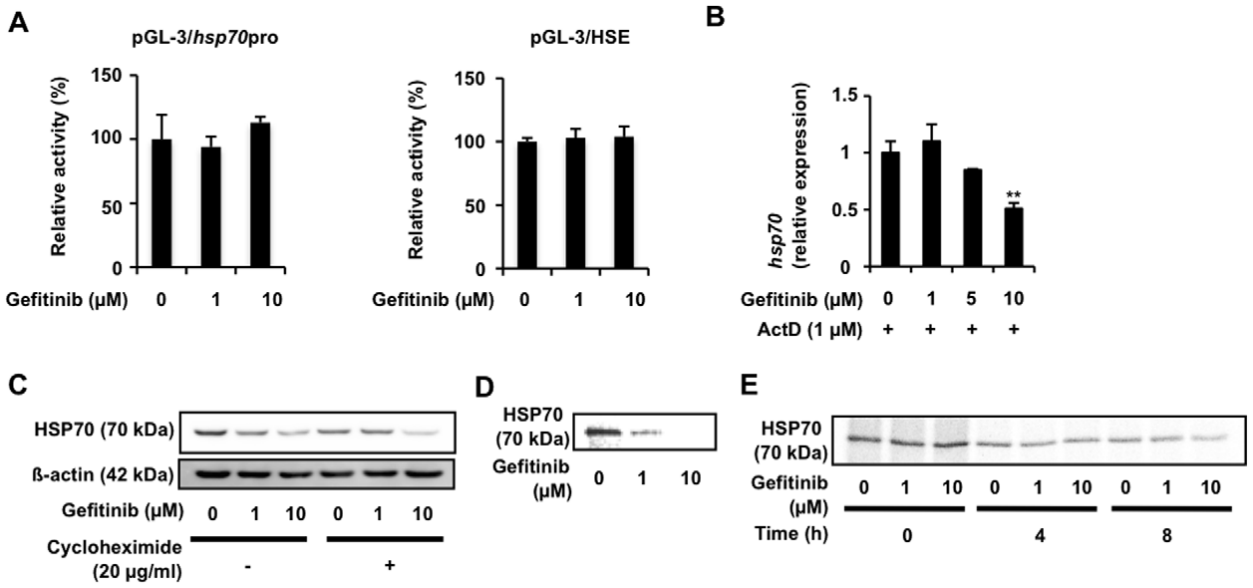
Translational regulation of expression of HSP70 by gefitinib. A549 cells were co-transfected with pRL-SV40 (internal control plasmid carrying the *R. reniformis luciferase* gene) and a pGL-3 derivative (pGL-3/*hsp70*pro or pGL-3/HSE) and cultured for 24 h. Cells were incubated with the indicated concentration of gefitinib for 24 h and *P. pyralis* luciferase activity was measured and normalized for *R. reniformis* luciferase activity. The 100% value of the *P. pyralis* luciferase activity is 6.9×10^4^ or 5.8×10^4^ units for pGL-3/*hsp70*pro or pGL-3/HSE, respectively (A). A549 cells were pre-incubated with 1 µg/ml actinomycin D (ActD) (B) or 20 µg/ml cycloheximide (C) for 1 h and further incubated for 8 h (B) or 24 h (C) with the indicated concentration of gefitinib (B, C). The mRNA (B) and protein (C) expression was monitored and is expressed as described in the legend of Fig. 1 A549 cells were pulse-labelled for 15 min with [^35^S]methionine and [^35^S]cysteine (D, E). Before the pulse-labelling, cells were incubated with the indicated concentration of gefitinib for 8 h (D). Pulse-labelled proteins were chased with excess amounts of Non-radioactively labeled methionine and cysteine for the indicated period in the presence of the indicated concentration of gefitinib (E). Labelled proteins were extracted, immunoprecipitated with antibody against HSP70, subjected to SDS-PAGE and autoradiographed (D, E). Values are mean ± S.D. (*n* = 3). **P<0.01.

To confirm this, a luciferase reporter assay was used to examine the effect of gefitinib on the promoter activity of *hsp70*. Treatment of cells with gefitinib did not affect the luciferase activity in cells carrying a reporter plasmid with an *hsp70* promoter or an HSE inserted upstream of the *luciferase* gene (Fig. 2A), suggesting that gefitinib does not affect the promoter activity of *hsp70*. Further to this, 1 µM gefitinib did not affect the *hsp70* mRNA level in cells pre-treated with actinomycin D, an inhibitor of transcription (Fig. 2B), suggesting that treatment of cells with 1 µM gefitinib does not affect *hsp70* mRNA stability. The results in Fig. 1A and B also suggest that treatment of cells with 10 µM gefitinib affects *hsp70* mRNA stability but not promoter activity.

We then tested whether gefitinib affects the translation and degradation of HSP70. As shown in Fig. 2C, the decrease in the level of HSP70 with 1 µM gefitinib was not observed in cells pre-treated with cycloheximide, an inhibitor of protein synthesis. Furthermore, a protein pulse-labelling experiment showed that the synthesis of HSP70 was inhibited in cells pre-treated with gefitinib (Fig. 2D). These results suggest that 1 µM gefitinib inhibits the translation of HSP70. On the other hand, the results of the pulse-chase experiment suggest that treatment of cells with 1 µM gefitinib does not affect the stability of HSP70; 1 µM gefitinib did not affect the level of labelled HSP70 after chase periods of 4 h or 8 h (Fig. 2E). The results in Fig. 2E also suggest that treatment of cells with 10 µM gefitinib stimulates the degradation of HSP70.

**Figure 3:**
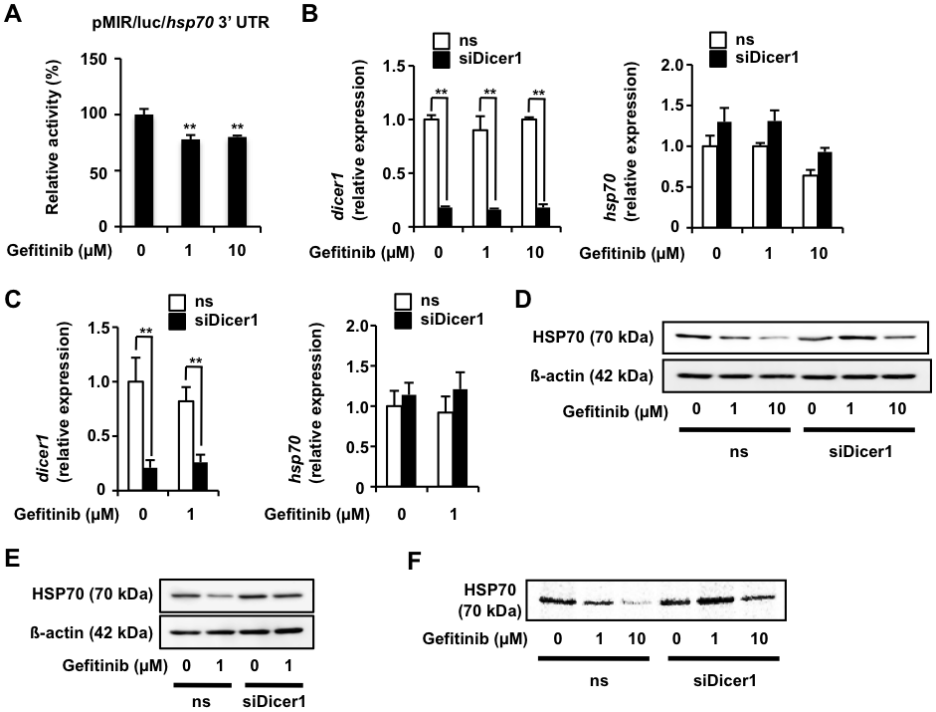
Contribution of miRNA to gefitinib-dependent suppression of expression of HSP70. A549 cells were co-transfected with pRL-SV40 and pMIR/luc/*hsp70* 3' UTR and cultured for 24 h. Cells were incubated with the indicated concentration of gefitinib for 24 h and luciferase reporter assay was done as described in the legend of Fig. 2. The 100% value of the *P. pyralis* luciferase activity is 1.2×10^5^ units (A). A549 cells were transfected with siRNA for Dicer1 (siDicer1) or non-specific siRNA (ns) (B-D). After 24 h, cells were incubated with the indicated concentration of gefitinib for 12 h (B), 24 h (C) or 8 h (D). The mRNA (B) and protein (C) expression was monitored and is expressed as described in the legend of Fig. 1. Pulse-labelling experiments were performed as described in the legend of Fig. 2 (D). Values shown are mean ± S.D. (*n* = 3). **P<0.01; n.s., not significant.

3' UTRs of genes play an important role in translational regulation, including that by miRNAs. Thus, we examined the contribution of this region to gefitinib-induced suppression of expression of HSP70 by a luciferase reporter assay. Treatment of cells with gefitinib significantly decreased the luciferase activity in cells carrying the reporter plasmid in which the 3' UTR of *hsp70* was inserted downstream of the *luciferase* gene (Fig. 3A), suggesting that 3' UTR-mediated modulation of HSP70 translation plays an important role in the gefitinib-induced suppression of HSP70 expression.

The contribution of miRNAs to gefitinib-induced suppression of HSP70 expression was then tested by use of siRNA for Dicer1, a protein essential for maturation of miRNAs. As shown in Fig. 3B, transfection of cells with siRNA for Dicer1 decreased the level of *Dicer1* mRNA, but not that of *hsp70*, suggesting that the miRNA system is not involved in the regulation of *hsp70* mRNA stability. The decrease in the level of HSP70 and inhibition of HSP70 protein synthesis in the presence of 1 µM gefitinib were not observed in cells transfected with siRNA for Dicer1 (Fig. 3C and D), suggesting that the 1 µM gefitinib-dependent inhibition of HSP70 translation is mediated by the miRNA system. However, the 10 µM gefitinib-dependent decrease in the level of HSP70 and inhibition of protein synthesis of HSP70 were observed even in cells transfected with siRNA for Dicer1 (Fig. 3C and D).

**Figure 4:**
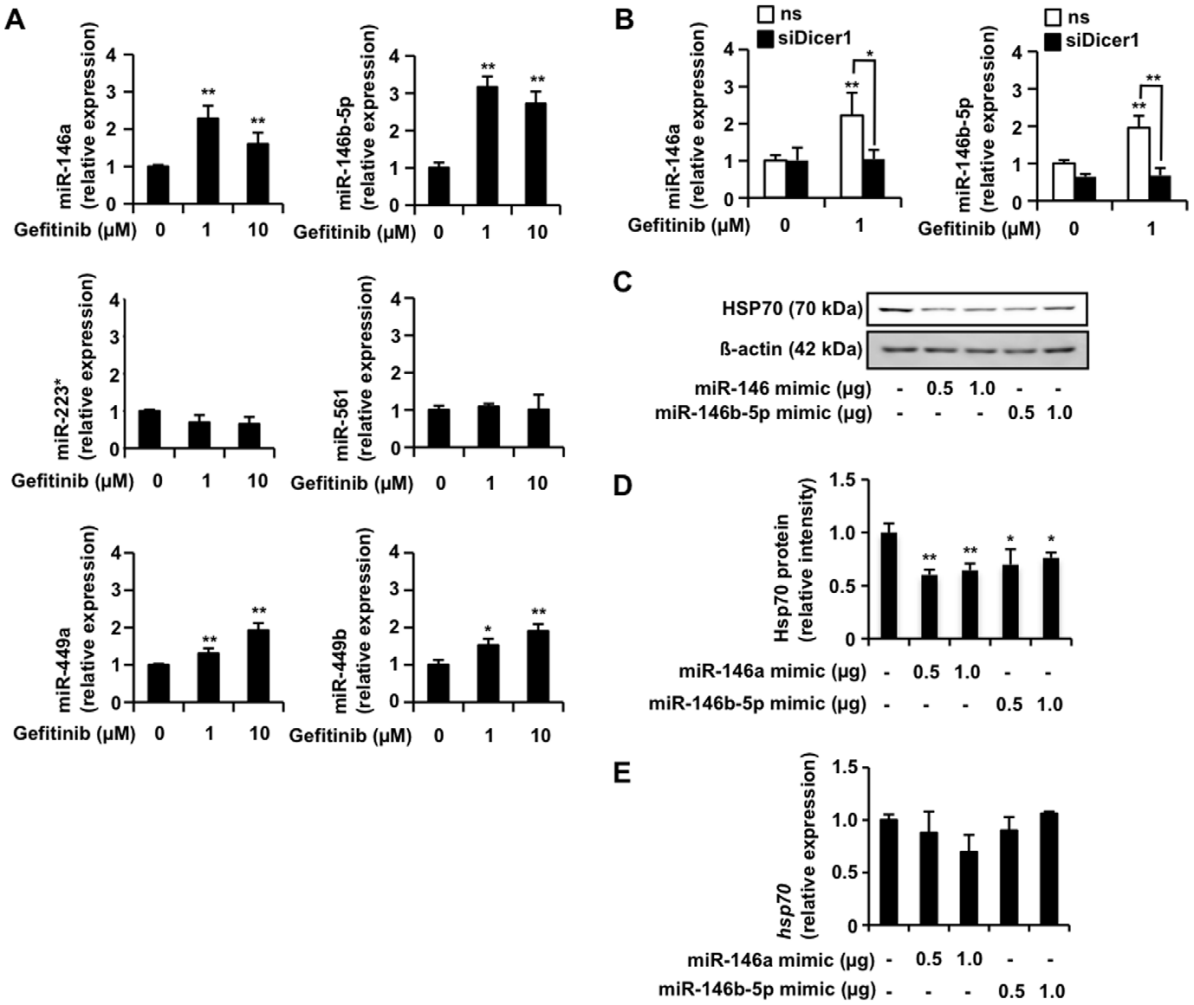
Identification of miRNAs involved in gefitinib-dependent suppression of expression of HSP70. A549 cells were incubated with the indicated concentration of gefitinib for 3 h (A). A549 cells were transfected with siRNA for Dicer1 (siDicer1) or non-specific siRNA (ns) and after 24 h were incubated with the indicated concentration of gefitinib for 3 h (B). A549 cells were transfected with the indicated amount ( µg/well) of miRNA mimic RNA fragments for 24 h (C, E). The RNA (A, B, E) and protein (C) expression was monitored and expressed as described in the legend of Fig. 1. The RUN44 non-coding RNA was used for normalization (A, B, E). The intensities of the HSP70 bands were determined and are expressed relative to the control (one of the gels is shown in C) (D). Values shown are mean ± S.D. (*n* = 3). **P<0.01; *P<0.05; n.s., not significant.

Next, using a database, we searched for miRNAs with sequences complementary to the 3' UTR of *hsp70.* Six candidate miRNAs were found (miR-146a, miR-146b-5b, miR-223*, miR-561, miR-449a and miR-449b). Real-time RT-PCR analysis revealed that among these miRNAs, the levels of miR-146a and miR-146b-5b clearly increased after treatment of cells with 1 µM gefitinib (we used experimental conditions of real-time RT-PCR under which only fully processed miRNA could be detected) (Fig. 4A). Furthermore, transfection of cells with siRNA for Dicer1 suppressed this gefitinib-dependent increase in the levels of these miRNAs (miR-146a, miR-146b-5b) (Fig. 4B) and transfection of cells with synthesized miR-146a and miR-146b-5b mimic RNA fragments decreased the level of HSP70 (Fig. 4C and D) but not that of *hsp70* mRNA (Fig. 4E). These results suggest that a gefitinib-dependent increase in the levels of miR-146a and miR-146b-5b is involved in gefitinib-dependent inhibition of HSP70 translation.

### Involvement of EGFR and JNK inhibition in gefitinib-dependent suppression of expression of HSP70

**Figure 5:**
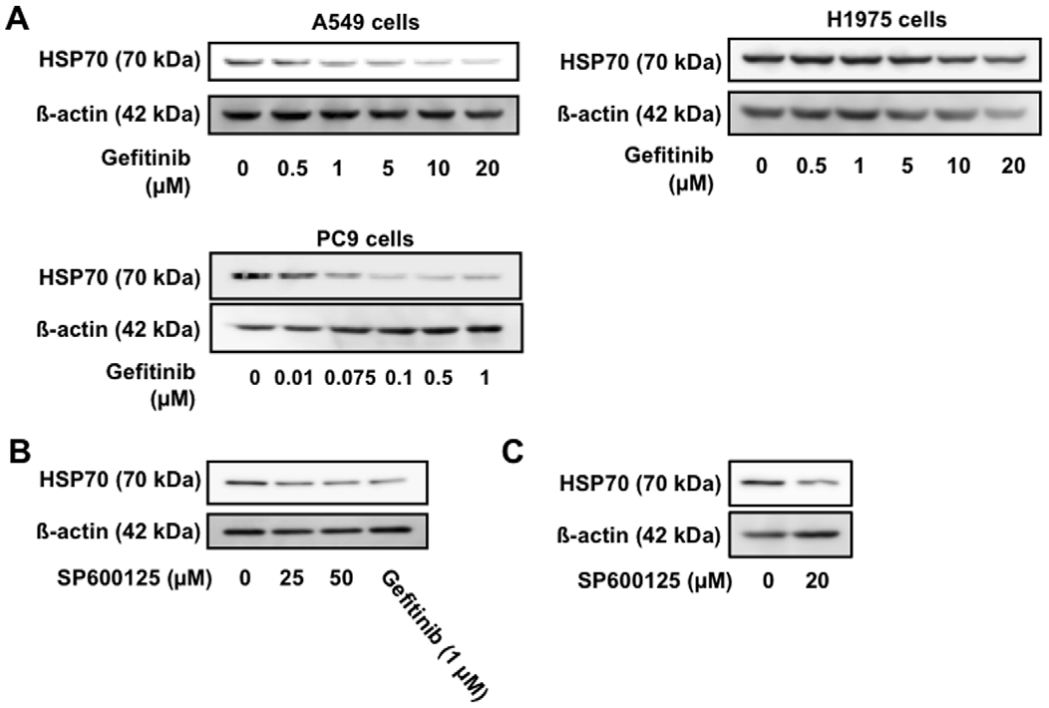
Involvement of inhibition of EGFR and JNK in gefitinib-dependent suppression of expression of HSP70. A549 (A-C) and H1975 and PC9 (A) cells were incubated with the indicated concentration of gefitinib (A, B) or SP600125 (B) for 24 h. Protein expression was monitored and is expressed as described in the legend of Fig. 1.

As described above, gefitinib inhibits self-phosphorylation of tyrosine residues in the cytosolic domains of EGFR. Thus, here we tested whether or not gefitinib suppresses expression of HSP70 through inhibition of the EGFR. To do this we used the cell lines H1975 and PC9 which are resistant or sensitive, respectively, to gefitinib-induced inhibition of the self-phosphorylation of EGFR and the resulting modulation of intracellular signal transduction due to mutations in the EGFR [43], [44]. As shown in Fig. 5A, the concentration of gefitinib required for suppression of HSP70 expression was higher and lower in H1975 cells and PC9 cells, respectively, than for A549 cells, suggesting that gefitinib suppresses expression of HSP70 through an inhibitory effect on the self-phosphorylation of EGFR.

Activation of the EFGR affects various cellular responses through modulation of intracellular signal transduction, such as activation of JNK and ERK [45], [46]. To test whether inhibition of JNK or ERK is involved in gefitinib-dependent suppression of HSP70 expression, we examined the effect of inhibitors of JNK and ERK, SP600125 and U0126, respectively, on the expression of HSP70. Treatment of cells with SP600125 (Fig. 5B) but not U0126 (data not shown) decreased the level of HSP70, suggesting that the inhibition of JNK is involved in the gefitinib-dependent suppression of HSP70 expression.

### Effect of gefitinib on bleomycin-induced pulmonary fibrosis

As described in the Introduction, we recently reported that expression of HSP70 protects against bleomycin-induced pulmonary fibrosis [35]. Therefore, the *in vitro* results outlined above imply that gefitinib exacerbates bleomycin-induced pulmonary fibrosis through suppression of HSP70 expression. To test this, we examined the effect of oral daily administration of gefitinib on the expression of HSP70 in the lungs. As shown in Fig. S2, this administration decreases the level of HSP70. The decrease became apparent at day 3 and was also observed at day 6 (Fig. S2). Therefore, to examine the effect of administration of gefitinib on bleomycin-induced pulmonary fibrosis, mice were orally administered gefitinib once per day for three days before receiving a single intratracheal administration of bleomycin; the administration of gefitinib was then continued, once every two days, for the following 14 days.

**Figure 6:**
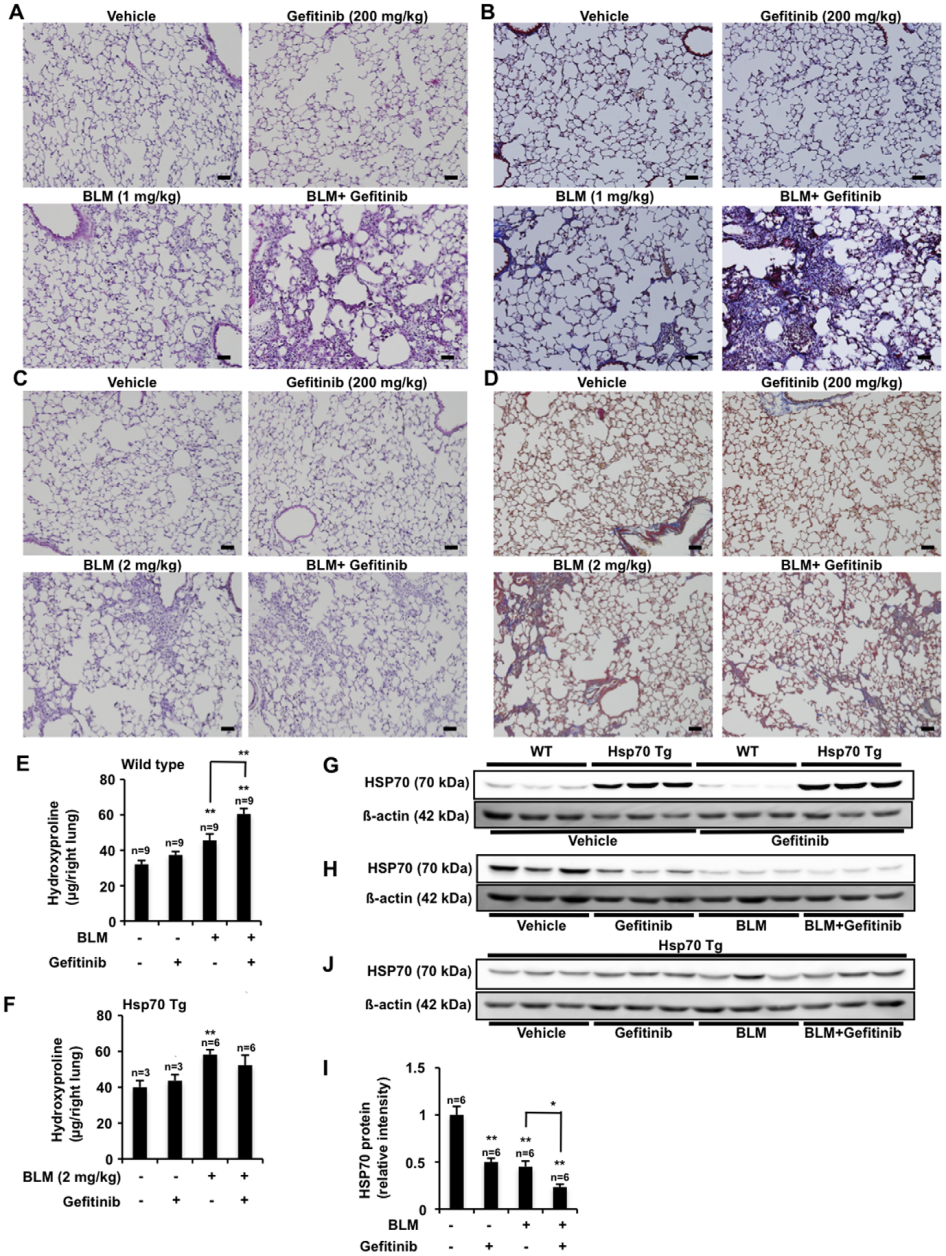
Effect of gefitinib on bleomycin-induced pulmonary fibrosis and pulmonary expression of HSP70. Wild-type mice (A, B, E, G, H) and transgenic mice expressing HSP70 (C, D, F, G, J) were orally administered gefitinib (200 mg/kg) or vehicle once per day for 3 days (from day 0 to day 2), then treated once only with or without 1 mg/kg (for wild-type mice) or 2 mg/kg (for the transgenic mice) bleomycin (BLM) (day 3). Mice were then orally administered gefitinib (200 mg/kg) or vehicle once per 2 days for 14 days (from day 3 to day 17). Sections of pulmonary tissue were prepared at day 17 and subjected to histological examination (H & E staining (A, C) or Masson's trichrome staining (B, D)) (scale bar, 50 µm) (A-D). Pulmonary hydroxyproline levels at day 17 were determined (E, F). Total protein was extracted from pulmonary tissues at day 17 and analyzed by immunoblotting with an antibody against HSP70 or actin (G, H, J). The intensities of the HSP70 bands were determined (one of the gels is shown in H) and are expressed relative to the control (I). Values are mean ± S.E.M. *P<0.05; **P<0.01; n.s., not significant.

Histopathological analysis of pulmonary tissue using hematoxylin and eosin (H & E) staining revealed that the simultaneous oral administration of gefitinib (200 mg/kg) and intratracheal administration of bleomycin (1 mg/kg) produced severe pulmonary damage (thickened and edematous alveolar walls and interstitium, and infiltration of leucocytes) (Fig. 6A). Administration of either gefitinib or bleomycin alone did not cause such clear-cut pulmonary damage (Fig. 6A). Masson's trichrome staining of collagen revealed that administration of bleomycin caused slight collagen deposition, an effect that was greatly exacerbated by simultaneous administration of gefitinib (Fig. 6B). The pulmonary hydroxyproline level (an indicator of collagen levels) was increased by administration of bleomycin alone, and this effect was further enhanced by the simultaneous administration of gefitinib (Fig. 6E). Under our experimental conditions, administration of gefitinib alone did not cause collagen deposition nor increase the level of pulmonary hydroxyproline (Fig. 6B and E).

We also performed similar experiments in transgenic mice expressing HSP70. Since these mice are resistant to bleomycin, we used a higher dose (2 mg/kg) of bleomycin than in the previously described experiments. As shown in Fig. 6C, D and F, the gefitinib-dependent enhancement of bleomycin-induced pulmonary damage, collagen deposition and increase in pulmonary hydroxyproline level were not clearly observed in the transgenic mice, suggesting that gefitinib exacerbates bleomycin-induced pulmonary fibrosis through the suppression of HSP70 expression.

To test this idea, we examined the effect of the administration of gefitinib and/or bleomycin on pulmonary expression of HSP70 in wild-type mice and transgenic mice expressing HSP70. To begin with, we confirmed the overexpression of HSP70 in the lungs of transgenic mice in both the presence and absence of gefitinib administration (Fig. 6G). As shown in Fig. 6H and I, in wild-type mice, administration of gefitinib decreased the pulmonary level of HSP70 in both the presence and absence of simultaneous bleomycin administration. Administration of bleomycin alone also decreased the pulmonary level of HSP70 (Fig. 6H and I). In contrast, such a gefitinib-dependent decrease in the pulmonary level of HSP70 was not observed clearly in transgenic mice expressing HSP70 in both the presence or absence of simultaneous administration of bleomycin (Fig. 6J). We also found that using a lower dose of gefitinib (100 mg/kg) neither exacerbated bleomycin-induced pulmonary fibrosis nor suppressed pulmonary expression of HSP70 (Fig. S3). These results suggest that administration of gefitinib exacerbates bleomycin-induced pulmonary fibrosis through suppression of HSP70 expression.

**Figure 7:**
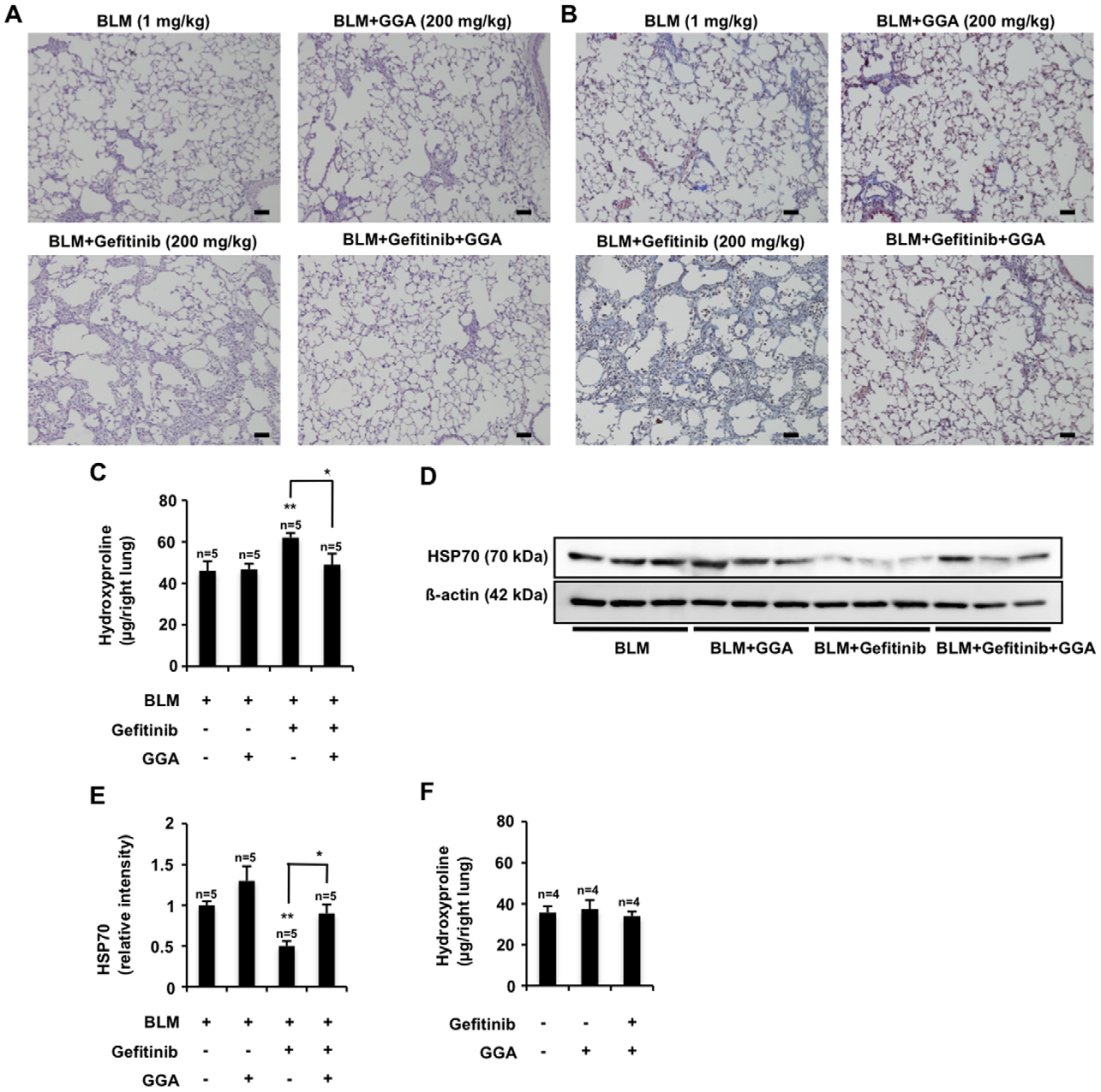
Effect of GGA on gefitinib-dependent exacerbation of pulmonary fibrosis and pulmonary expression of HSP70. Wild-type mice were orally administered gefitinib (200 mg/kg) and/or GGA (200 mg/kg) once per day for 3 days (from day 0 to day 2), then treated once only with 1 mg/kg bleomycin (BLM) (day 3). Mice were then orally administered gefitinib (200 mg/kg) and/or GGA (200 mg/kg) once per 2 days for 14 days (from day 3 to day 17). Sections of pulmonary tissue were prepared at day 17 and subjected to histological examination (H & E staining (A) or Masson's trichrome staining (B)) (scale bar, 50 µm). The pulmonary hydroxyproline levels were determined at day 17 (C). Total protein was extracted from pulmonary tissues at day 17 and analyzed and expressed as described in the legend of Fig. 6 (D, E). Values are mean ± S.E.M. **P<0.01; *P<0.05.

For further confirmation of this idea, we examined the effect of co-administration of GGA. As shown in Fig. 7A-C, co-administration of GGA clearly suppressed the gefitinib-dependent enhancement of bleomycin-induced pulmonary tissue damage, collagen deposition and increases in the pulmonary hydroxyproline level in wild-type mice. We also examined the effect of GGA on the expression of HSP70 in the lung. As shown in Fig. 7D and E, co-administration of GGA recovered the gefitinib-suppressed expression of HSP70 in the lung, suggesting that GGA suppresses the gefitinib-dependent exacerbation of bleomycin-induced pulmonary fibrosis by inducing the recovery of HSP70 expression.

## Discussion

Because the molecular mechanism governing drug-induced ILD (interstitial pneumonia associated with pulmonary fibrosis) is unknown, a suitable animal model is not available at present. Consequently, neither a clinical protocol for the treatment of drug-induced ILD nor an assay system to eliminate candidate drugs with the potential to cause this type of side effect has been established. We recently reported that A771726, an active metabolite of leflunomide, induced EMT of lung epithelial cells both *in vitro* and *in vivo*, and that administration of leflunomide exacerbated bleomycin-induced pulmonary fibrosis, suggesting that the leflunomide-dependent exacerbation of bleomycin-induced pulmonary fibrosis is mediated by the stimulation of EMT of lung epithelial cells. We proposed that examination of the EMT-inducing ability of candidate drugs is useful for screening to eliminate those with the potential side effect of inducing pulmonary fibrosis. We also found that leflunomide-dependent exacerbation of bleomycin-induced pulmonary fibrosis is ameliorated by the simultaneous intratracheal administration of uridine, which suppresses the A771726-dependent induction of EMT *in vitro*, and propose that this administration is beneficial for the treatment of leflunomide-induced pulmonary fibrosis in humans. In the present study, we followed a similar strategy, focusing on the expression of HSP70, because we had recently reported that expression of HSP70 protects against bleomycin-induced pulmonary fibrosis through cytoprotective and anti-inflammatory effects and, by inhibiting the production of TGF-β1 and TGF-β1-dependent EMT of lung epithelial cells [35].

We have found that gefitinib suppresses the expression of HSP70 *in vitro*. The concentration of gefitinib used (1 µM) is similar to that obtained in plasma when administered at therapeutic levels (about 1-2 µM) [47]. Thus, we considered that the suppression of HSP70 expression by gefitinib is clinically relevant and investigated the molecular mechanism underlying this effect. We concluded that treatment of cells with gefitinib (1 µM) decreases the level of HSP70 through inhibition of translation based on observations that (i) the gefitinib-dependent decrease in the level of HSP70 was suppressed by an inhibitor of translation, (ii) the translation of HSP70, measured by pulse-labelling experiments, was inhibited by treatment of cells with gefitinib, and (iii) *hsp70* promoter activity, the level and stability of *hsp70* mRNA and the stability of HSP70 were all unaffected by treatment of cells with gefitinib. Furthermore, we suggested that two miRNAs (miR-146a and miR-146b-5p) are involved in this gefitinib*-*dependent suppression of HSP70 translation; this was based on our observations that (i) the introduction of the 3' UTR of *hsp70* into a luciferase reporter plasmid caused a gefitinib*-*dependent decrease in luciferase activity, (ii) the gefitinib*-*dependent suppression of HSP70 translation was not observed in cells transfected with siRNA for Dicer1, and (iii) gefitinib increased the levels of miR-146a and miR-146b-5p, both of which have the ability to decrease the level of HSP70. Since for various cancer cells and tissues, down-regulation of expression of these miRNAs and up-regulation of HSP70 and the contribution of these to cancer progression have been reported [48], [49], [50], the results of this study suggest the latter up-regulation is caused by the former down-regulation.

A decrease in the level of HSP70 was also observed in cells treated with 10 µM gefitinib; however, the mechanism governing this decrease seems to be different from that seen with 1 µM gefitinib, because treatment of cells with 10 µM gefitinib caused a decrease in the level of *hsp70* mRNA and in the stability of both *hsp70* mRNA and HSP70. We consider that this high concentration of gefitinib pleiotropically affects various cellular reactions.

After binding EGF, the EGFR is self-phosphorylated and transduces the signal via various mechanisms, including the activation of JNK. Here, we have found that mutations in the EGFR that alter the concentration of gefitinib required for inhibition of EGFR self-phosphorylation affect the gefitinib-dependent decrease in the level of HSP70. Furthermore, an inhibitor of JNK caused a decrease in the level of HSP70. These results suggest that inhibition of EGFR self-phosphorylation and the resulting inhibition of JNK are involved in the gefitinib-dependent decrease in the level of HSP70. However, the manner by which inhibition of JNK suppresses translation of HSP70 remains unclear.

Administration of gefitinib alone did not produce pulmonary fibrosis under our experimental conditions. As one of the risk factors for gefitinib-induced ILD is pre-existing pulmonary fibrosis, we hypothesized that gefitinib stimulates pulmonary fibrosis in the presence of other fibrosis-inducing stimuli. In fact, we found that administration of gefitinib stimulates bleomycin-induced pulmonary fibrosis. Interestingly, this gefitinib-dependent stimulation was not observed so clearly in transgenic mice expressing HSP70, suggesting that expression of HSP70 is involved in the gefitinib-induced exacerbation of bleomycin-induced pulmonary fibrosis. We found that administration of gefitinib suppressed the pulmonary expression of HSP70 and that this suppression was not observed in the transgenic mice expressing HSP70. Taken together, our findings suggest that gefitinib exacerbates bleomycin-induced pulmonary fibrosis through the suppression of HSP70 expression. This finding is an important step towards elucidating the molecular mechanism of drug-induced ILD, as well as the mechanism governing the ethnic differences in susceptibility to ILD induced by the drug. It is possible that the susceptible phenotype of Japanese patients is due to a specific polymorphism in the *hsp70* and *hsf1* genes and in other genes related to HSP70. Also, examination of the effect of candidate drugs on the expression of HSP70 *in vitro* could be used for screening to eliminate those drugs with the potential to induce ILD. Furthermore, exacerbation of bleomycin-induced pulmonary fibrosis was observed with leflunomide [7] and gefitinib (this study), suggesting this model can be used as an animal model of drug-induced pulmonary fibrosis and for screening to eliminate candidate drugs with the potential to induce ILD.

As described above, it was recently reported that administration of GGA ameliorates bleomycin-induced pulmonary fibrosis [51], a result that we confirmed in a previous paper [35], In this study, we found that administration of GGA suppresses the gefitinib-dependent exacerbation of bleomycin-induced pulmonary fibrosis. We consider that this suppression is due to the recovery of HSP70 expression in the lung. As described in the Introduction, a treatment for drug-induced ILD has not been established. We believe that GGA could be beneficial for the treatment of gefitinib-induced ILD, given that the safety of GGA has already been shown clinically.

## Supporting Information

Figure S1 **Effects of drugs known to induce ILD clinically on expression of HSP70.** A549 cells were incubated with the indicated concentration of A771726, amiodarone (AMD), gefitinib or imatinib for 24 h. Whole cell extracts were analyzed by immunoblotting with an antibody against HSP70 or β-actin.
(TIFF)

Figure S2 **Time course profile for gefitinib-dependent suppression of expression of HSP70** ***in vivo*** **.** Mice were orally administered gefitinib (200 mg/kg) or vehicle once per day for the indicated periods. Total protein was extracted from pulmonary tissues and protein expression was monitored by immunoblotting with an antibody against HSP70 or β-actin.
(TIFF)

Figure S3 **Effect of low dose of gefitinib on bleomycin-induced pulmonary fibrosis and pulmonary expression of HSP70.** The effect of a low dose of gefitinib (100 mg/kg) on the expression of HSP70 in the lung (A) and pulmonary hydroxyproline levels (B) were monitored as described in the legend of Fig. 6. Values are mean ± S.E.M.
(TIFF)
