# Supplementary figures and images for: Suppression of Expression of Heat Shock Protein 70 by Gefitinib and Its Contribution to Pulmonary Fibrosis
Source: PLoS One. 2011 Nov 9;6(11):e27296. doi: 10.1371/journal.pone.0027296 (PMC3212557; doi:10.1371/journal.pone.0027296)

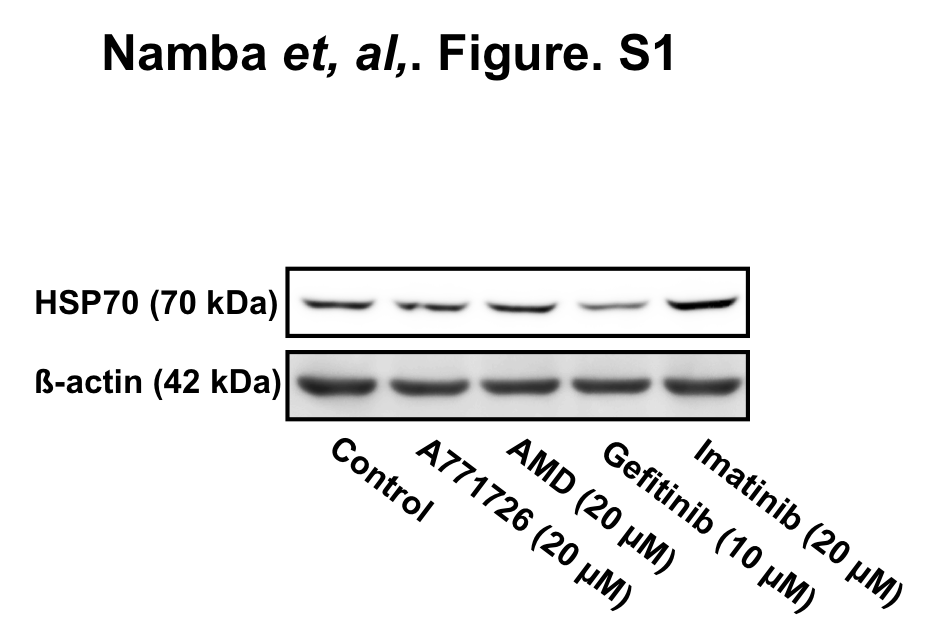

Supplement: Figure S1 — Effects of drugs known to induce ILD clinically on expression of HSP70. A549 cells were incubated with the indicated concentration of A771726, amiodarone (AMD), gefitinib or imatinib for 24 h. Whole cell extracts were analyzed by immunoblotting with an antibody against HSP70 or β-actin. (TIFF) [file pone.0027296.s001.tif]

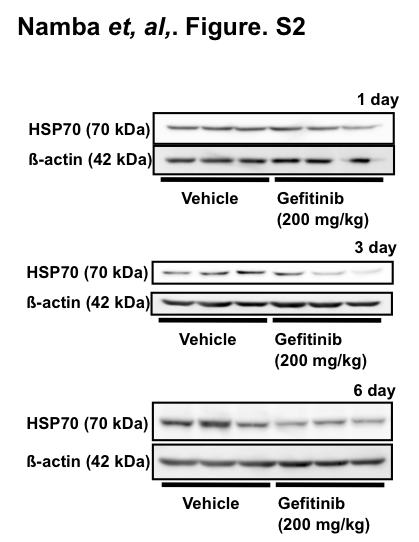

Supplement: Figure S2 — Time course profile for gefitinib-dependent suppression of expression of HSP70 in vivo . Mice were orally administered gefitinib (200 mg/kg) or vehicle once per day for the indicated periods. Total protein was extracted from pulmonary tissues and protein expression was monitored by immunoblotting with an antibody against HSP70 or β-actin. (TIFF) [file pone.0027296.s002.tif]

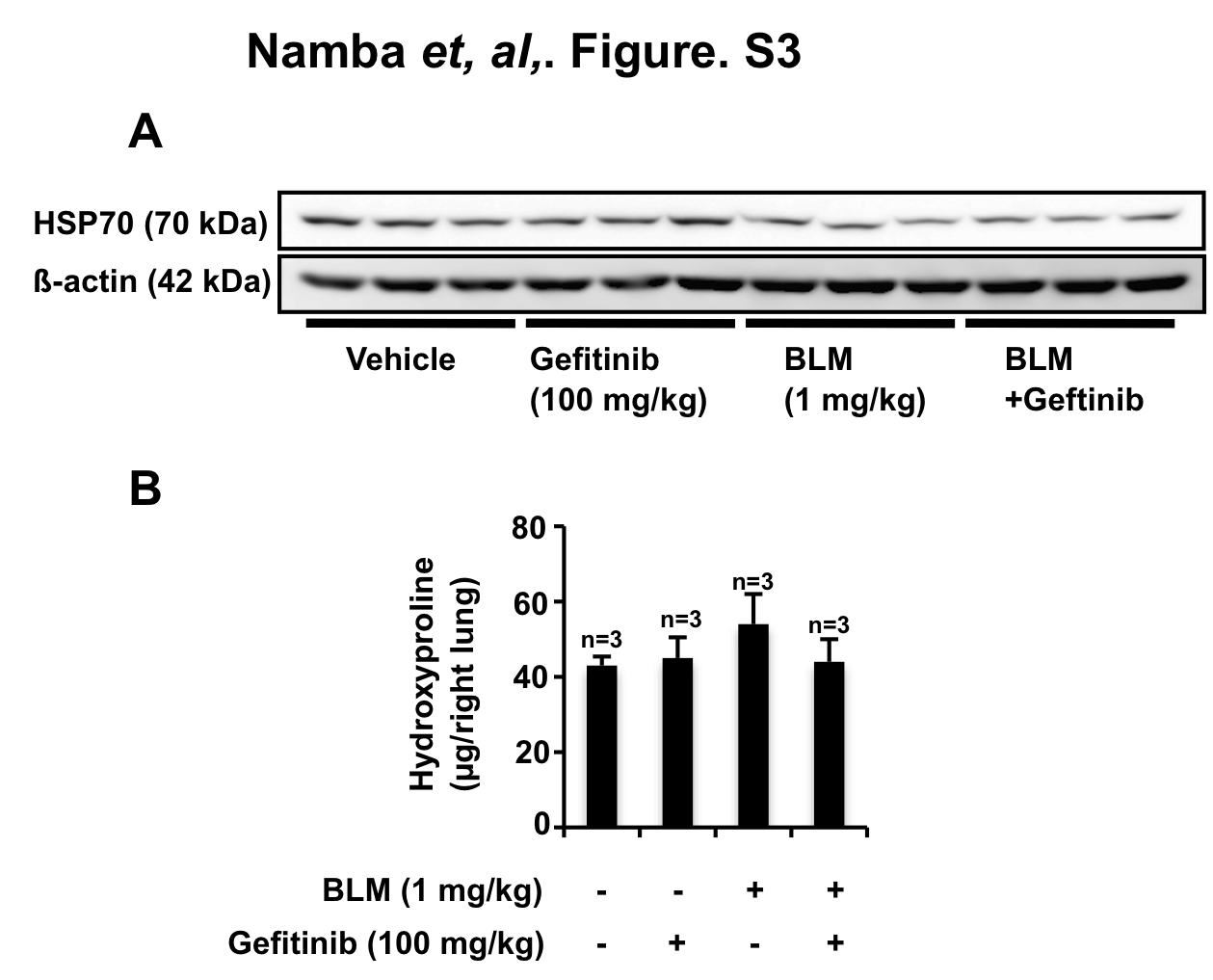

Supplement: Figure S3 — Effect of low dose of gefitinib on bleomycin-induced pulmonary fibrosis and pulmonary expression of HSP70. The effect of a low dose of gefitinib (100 mg/kg) on the expression of HSP70 in the lung (A) and pulmonary hydroxyproline levels (B) were monitored as described in the legend of Fig. 6. Values are mean ± S.E.M. (TIFF) [file pone.0027296.s003.tif]
